# Intravenous lipomas of head and neck: an exceptional entity and its clinical implications

**DOI:** 10.3389/fneur.2024.1447960

**Published:** 2024-08-19

**Authors:** Verónica Fernández-Alvarez, Miriam Linares-Sánchez, Antti A. Mäkitie, Carlos Suárez, Eelco de Bree, Alexander Karatzanis, Robert P. Takes, Primoz Strojan, Alessandra Rinaldo, Alfio Ferlito

**Affiliations:** ^1^Department of Vascular and Endovascular Surgery, Torrecardenas University Hospital, Almeria, Spain; ^2^Department of Vascular and Endovascular Surgery, Cabueñes University Hospital, Gijon, Spain; ^3^Department of Otorhinolaryngology – Head and Neck Surgery, Research Program in Systems Oncology, University of Helsinki and Helsinki University Hospital, Helsinki, Finland; ^4^Department of Otorhinolaryngology, University of Oviedo, Oviedo, Spain; ^5^Department of Surgical Oncology, Medical School of Crete University Hospital, Heraklion, Greece; ^6^Department of Otorhinolaryngology, Medical School of Crete University Hospital, Heraklion, Greece; ^7^Department of Otorhinolaryngology and Head and Neck Surgery, Radboud University Medical Center, Nijmegen, Netherlands; ^8^Department of Radiotherapy, Institute of Oncology, Ljubljana, Slovenia; ^9^ENT Unit, Policlinico Città di Udine, Udine, Italy; ^10^Coordinator of the International Head and Neck Scientific Group, Padua, Italy

**Keywords:** lipoma, intravenous lipoma, primary venous tumor, superior vena cava, head and neck

## Abstract

Intravenous lipomas (IVLs) of the head and neck are uncommon benign tumors that develop within the venous walls, often detected incidentally during imaging for unrelated issues. While usually asymptomatic, these IVLs can cause congestive venous symptoms like swelling, paresthesia or pain in the head and neck and upper limbs, or even venous thromboembolism. The precise diagnosis of IVLs is predominantly achieved through computed tomography (CT) and magnetic resonance imaging (MRI), with CT being the most frequently used method. Symptomatic patients generally undergo open surgery with excision of the IVL followed by venous reconstruction, which has shown safe and effective outcomes. However, the management of asymptomatic IVLs remains controversial due to the limited number of reported cases. Despite this, there is a notable trend toward recommending surgical removal of IVLs to prevent complications and rule out malignancy, driven by the challenges of differentiating IVLs from malignant tumors using imaging alone. This review highlights the key differential imaging characteristics of IVLs and the main surgical techniques to remove the tumor and repair the vascular defect. Further research is necessary to establish a robust, evidence-based approach for treating asymptomatic IVLs, balancing the risks of surgery against the potential for future complications.

## 1 Introduction

Lipomatous tumors in the head and neck region are rare, exhibiting a wide spectrum of biological behaviors, from completely benign to malignant with metastatic potential. Ordinary lipoma, the most prevalent benign tumor in adults, constitutes approximately 30–50% of all soft tissue tumors and predominantly occur in the subcutaneous tissue of the upper body and proximal extremities (1–4), with around 13–17% located in the head and neck region (1). However, intravenous lipomas (IVLs) are extremely rare primary venous tumors originating intravascularly. While these lipomas are most commonly found in the heart (5), their occurrence in central veins is exceptionally uncommon (6). The inferior vena cava (IVC) is the most common location for IVLs, with few cases reported in the head and neck region (6). Many IVLs are asymptomatic and are often discovered during imaging studies for other conditions, while others mimic deep vein thrombosis by causing compressive or occlusive symptoms (5, 7–9). Although IVLs have a favorable prognosis, their clinical course is not well understood. Due to the scarcity of reported cases, the indication for surgical intervention is controversial, particularly in asymptomatic patients, given the uncertain risk of thrombus formation, venous occlusion, and embolization. The aim of this mini-review is to clarify the natural history and optimal management strategies for IVL, thereby enhancing clinical decision-making and patient outcomes. Key points for imaging diagnosis are summarized, surgical techniques are briefly outlined, and associated patient outcomes are presented.

## 2 Literature review, primary anatomical locations, and demographic trends

A literature search was conducted across three major databases (PubMed, Elsevier, Google Scholar) using the terms “lipoma” AND (“intravascular” OR “intravenous” OR “superior vena cava” OR “brachiocephalic” OR “subclavian” OR “jugular”). If additional studies were identified from the references of previously published reviews, they were included in the analysis.

Only 24 cases of IVL were identified until May 2024 during the above literature search.

The majority of reported IVL cases in the head and neck region involved the superior vena cava (SVC), often extending to the brachiocephalic, subclavian or jugular veins (2, 4, 6–17). IVLs may also occur within the SVC without extension to adjacent veins (18–22). Additionally, isolated cases of jugular (5) or brachiocephalic vein lipomas (23–27) have also been documented. With the exception of cases reported by Kamdar et al. (11) and Fung (27), right-sided veins are typically involved. Regarding gender distribution, IVLs exhibited a male predominance with a ratio of 3:1 compared to females, while they are detected at an earlier age in women (mean age: 53.2 years, range: 39–70 years) compared to men (mean age: 58.5 years, range: 47–73 years) (2, 4–27). The occurrence rate of IVLs is unknown but it was estimated to be found, as an incidental finding, in 0.35–0.5% among the general population undergoing contrast-enhanced CT (4, 28).

Table 1 summarizes the case reports from the literature describing IVLs involving the SVC and cervical veins.

**Table 1:** Cases reported from the literature describing IVLs involving the SCV and cervical veins.

Author (year)	Age Gender	Location	Symptoms	Imaging studies	Size (mm)	Surgical repair
Vinnicombe et al. (1994) (6)	42/F	R BCV SVC	Fatigue, facial and hand edema	CT: rounded mass of fat attenuation reducing the lumen of R BCV and SVC. Venography: Lobulated filling defect, widening SVC.	100×50 ×50	NE
Thorogood et al. (1996) (8)	73/M	R BCV SVC	No	CT: mass of fat density within the lumen of the SVC and the R BCV. MRI T1-weighted: high signal mass. STIR sequence confirmed the fatty nature.	NE	No excision
Trabut et al. (1999) (19)	55/M	SVC	No	CT: findings not specified.	NE	NE
Lomeo et al. (2007) (4)	60/M	R SCV SVC	No	CT: Extravascular fatty mass adjacent to the R SCV with an intravascular SVC component. Echocardiography: mass adjacent to the SVC. Duplex US: no sign of thrombosis in the R SCV/SVC.	100	Direct primary suture
Moore et al. (2008) (23)	58/M	R BCV	No	CT: filling defect with fatty attenuation within the R BCV. MRI: confirmed fatty nature of the mass.	NE	No excision
Ryu et al. (2009) (10)	47/M	R SCV R BCV	No	CT: oval-shaped mass with fat attenuation. MRI T1 weighted: fatty mass based on the R SCV growing into the R BCV.	10×35	No excision
Kamdar et al. (2009) (11)	63/M	L IJV SVC	No	CT: fatty mass partially occluding L IJV and extending into the SCV. MRI T1-weighted: hyperintense mass that loses signal with fat saturation.	NE	Ligation of IJV
Mordant et al. (2010) (12)	55/F	R SCV R BCV SVC	No	CT: Intraluminal nonenhancing tumor. MRI: fatty intravascular lesion. Venography: total occlusion of the R SCV and BCP and SVC with abnormal collaterals.	90×50	End-to-end anastomosis between BCV and SVC
Bravi et al. (2012) (18)	63/M	R SCV SVC	Left-sided abdominal and right shoulder pain	CT: fatty mass within the lumen of SVC extending from right atrium to R SCV. MRI: uniform signal drop on fat-suppressed sequences. Echocardiogram: dilatated right atrium and a filling defect in the final portion of SVC.	130	Pericardial patch
Santos et al. (2012) (13)	47/F	R BCV SVC	No	CT: nonenhancing intraluminal polypoid mass with fat attenuation from the R BCV to SVC.	NE	No excision
Lococo et al. (2013) (24)	61/M	R BCV	No	CT: well-defined mass of fat density in the thoracic inlet space which invaded the R BCV.	41×23	PTFE patch
Yoon et al. (2013) (5)	39/F	R IJV	No	CT: fat attenuation mass in the caudal R IJV. Dupplex US: loosely attached, mobile, well-circumscribed hypoechoic mass.	10×10 ×12	Oversewn of both IJV ends
Iqbal et al. (2014) (25)	51/M	R BCV	No	CT: fatty mass (−129 HU). MRI: intravascular lipoma with no signs of malignancy. Duplex US: no evidence of venous stasis or compression.	13	No excision
Concatto et al. (2015) (20)	58/M	SVC	No	CT: hypodense elongated lesion with fat density within the SVC. MRI: confirmed the fatty nature of the lesion.	110	NE
Tanyeli et al. (2015) (21)	48/M	SVC	Right upper extremity swelling and paresthesia	CT: lesion of fat density within the SVC. MRI: intraluminal mass, causing enlargement and partial occlusion of the SVC.	50×20	NE
Vetrhus et al. (2017) (26)	60/F	R BCV	No	CT: filling defect with fatty attenuation within the R BCV.	17x8x 12	No excision
Wahab et al. (2017) (22)	70/F	SVC	No	MRI: filling defect at the junction of the RA and SVC. TEE: partially obstructing round, echogenic mass at the SVC and RA junction.	26 × 16 × 16	NE
Beliaev et al. (2019) (7)	49/F	R IJV R SCV SVC	No	CT: low density mass with smooth contours in the SVC, attached to the junction of the R SCV and IJV by a narrow stalk.	95×25	Direct primary suture
Elen et al. (2019) (14) Soetisna et al. (2022) (15)	54/M	SVC RA	No	CT: elongated lesion with low density (−102 HU) arised from SVC to RA. MRI: big capsulated mass, hyperintense at T1-weighted image but hypointense at fat suppression technique, with no enhancenment. Ecocardiography: large mass at RA.	120×50 ×40	Direct primary suture
Sundaram et al. (2020) (16)	58/M	R IJV R BCV SVC	Facial edema and right upper extremity venous congestion	CT: intraluminal mass in the IJV and BCV extending into SVC. Dupplex US: large pedunculated hyperechoic mass attached to R IJV and extending to R BCV. Diminished flow in the IJV.	50	Direct primary suture
Podobed (17) (2021)	53/F	R BCV SVC	Facial and right hand edema	CT: hypodense elongated lesion with fat density within the SVC.	NE	NE
Knop et al. (9) (2021)	59/M	R IJV R BCV SVC	No	CT: low density intraluminal mass R BCV extending to IJV and SVC.	NE	No excision
Cohen et al. (2) (2023)	64/F	R BCV SVC	No	CT: low density mass in the SVC extending to the R BCV with mild enhancement after contrast administration. MRI: fatty encapsulated tumor conditioning filling defect in SVC and R BCV.	76	Pericardial patch
Fung (2023) (27)	70/M	L BCV	No	CT: well-circumscribed hypodense lesion in the L BCV with fat density.	10×8	No excision

R BCV, right brachiocephalic vein; L BCV, left brachiocephalic vein; SVC, superior vena cava; R SCV, right subclavian vein; R IJV, right internal jugular vein; L IJV, left internal jugular vein; RA, right atrium; PTFE, polytetrafluoroethylene; TEE, transesophageal echocardiogram; US, ultrasound; NE, not specified.

## 3 Pathophysiological mechanisms and potential contributing factors

The risk factors for IVLs remain somewhat unclear due to the limited literature on this condition and the absence of experimental data. However, several authors have suggested that obesity, liver cirrhosis, hepatic tumors, renal angiomyolipomas, direct venous injury, and prolonged corticosteroid use may be associated with the presence of IVLs (7, 13, 29, 30).

Genetic factors are believed to play a significant role. However, while approximately 7–14% of patients with ordinary lipomas develop multiple lipomas, familial multiple lipomas are observed in about 30% of cases (1), suggesting a hereditary predisposition, only Thorogood et al. (8) have reported a case of SVC IVL associated with multiple cutaneous lipomas.

Santos et al. (13) have proposed a hypothesis regarding the potential role of immunologic phenomena associated with sarcoidosis, which could impact the structural components of vessel walls. This hypothesis is based on the plausible differentiation of vascular wall-resident multipotent stem cells, including mesenchymal stem cells, into adipocytes. Despite the few adipocytes typically present in the venous media layer, according to their assumption, these factors may contribute to the abnormal growth of fatty tissue within veins.

## 4 Natural history of IVLs and variants

### 4.1 Natural progression of IVLs

Several hypotheses have been proposed about the origin of IVLs. Initially, IVLs were believed to originate within the vein wall, with potential expansion into the vascular lumen or extraluminal invasion into surrounding adipose tissue (2, 6, 9, 13, 22). The first documented case of an IVL dates to 1962, reported by Tsardakas, involving the endothelium of the saphenous vein (31). However, subsequent reports by other authors have depicted cases where lipomas exhibited both intravascular and extravascular components (4, 5, 24). This duality has prompted the formulation of two theories to elucidate this peculiar presentation, positing intraluminal origin from the vein wall or extraluminal origin via invagination of adjacent adipose tissue (13). According to the former theory, the tumor grows into the vein wall protruding both internally and externally (4, 13, 23, 24, 29, 30, 32). The latter hypothesis suggests a perivascular tissue origin, infiltration into the vein wall, and subsequent protrusion into the lumen. Lomeo et al. (4) provided a detailed description of how the tumor arose from perivascular tissue and then intruded into the vein by invagination without infiltrating the wall. The CT scan revealed that initially, the mass was located outside the right subclavian vein; however, after a 2 cm distance, it traversed the venous wall and positioned itself within the vein lumen as it approached the SVC. The transmural vascular invasion of the IVL into the vein was described as a dumbbell-shaped extension of the tumor (24).

### 4.2 Pericaval fat collections as normal variants

Concerning fat mass-like lesions, localization adjacent to or projecting into the subdiaphragmatic portion of the intrahepatic IVC is recognized as a normal variant by some authors (28). Miyake et al. (28) first described these focal fat collections in 1992, found in 0.5% of 2,227 patients undergoing a CT examination. Subsequently, Perry et al. (30) reported similar findings in seven cases, terming them “lipomas.” Although these masses appeared intraluminal due to their acute angle with respect to the cava wall, speculation persisted regarding their external origin (30). Han et al. (33) attributed the appearance of pericaval fat collections to rightward angulation of the IVC and narrowing of the intrahepatic IVC. The stability of fat collections on follow-up CT scans in the majority of reported cases supports the notion that these findings likely represent entirely extraluminal lesions mimicking intraluminal masses. Thus, they are considered unusual but normal variants with no clinical significance, negating the need for patient follow-up (34).

## 5 Clinical presentation: how IVLs show up

IVLs are often asymptomatic and are commonly detected incidentally during imaging studies conducted for unrelated reasons (8, 9). When symptoms do occur, they are usually attributable to the location and tumor’s size, resulting in venous thrombosis, obstructive venous symptoms, or mediastinal syndromes due to compressive effects (6, 21, 23). Although SVC lipomas or IVLs in the head and neck region are rare compared to IVC counterparts, they may produce symptoms related to venous obstruction within the SVC drainage territory. This can manifest as SVC syndrome, swelling in the head, face, neck, and arms, as well as upper limb paresthesia, shoulder pain, and venous thromboembolism (6, 7, 12, 15, 17, 18, 21). In the literature review, only 21% of cases were associated with symptoms (6, 16–18, 21). Among them, facial and upper extremity edema have been reported as the main symptom of head and neck IVLs.

In cases involving larger IVLs within the SVC, there have been reports of intraluminal thrombus formation, such as the case documented by Bravi et al. (18) where an IVL within the SVC led to a subtotal occlusion extending into the right atrium, accompanied by thrombosis of the suprahepatic IVC and the portal vein. Similarly, Lococo et al. (24) reported a case of a fatty neoplastic thrombus completely occluding the brachiocephalic vein. It is postulated that the subsequent turbulent flow may promote thrombus formation and occlusion (18, 30).

## 6 Diagnostic imaging of IVLs and histopathologic findings

### 6.1 Key imaging features of IVLs

IVLs can be diagnosed using various imaging modalities, including sonography, CT, MRI, transthoracic/transesophageal echocardiogram, or venography (16). Among reported cases of IVLs in the head and neck region, CT and MRI are commonly employed for characterizing the mass (1).

**Figure 1:**
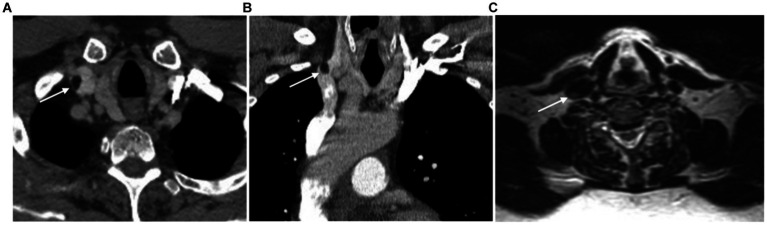
Axial **(A)** and coronal **(B)** images of a CT scan of the chest, revealing a 10 mm, well-defined, hypodense mass at the right jugulosubclavian venous confluence (white arrows). This lesion is consistent with the characteristics of an intravascular lipoma, exhibiting fat attenuation (-97 HU) and no evidence of significant contrast enhancement. On an axial T2-weighted MRI sequence without fat saturation **(C)**, the lipoma appears as a hyperintense, homogeneous mass.

Upon examination of chest X-rays, the identification of a widened superior mediastinum may signal the presence of a SVC IVL, as documented by Vinnicombe et al. (6). Sonographically, IVLs typically present as intraluminal hyperechoic masses with or without thin septa. Duplex venous sonography can show a patent vein or diminished flow if the lipoma is causing near obstruction (4, 9, 16, 25). However, MRI and CT are the most reliable imaging techniques for confidently identifying adipose tissue in these lesions (1). CT imaging may depict a well-defined, hypoattenuating intraluminal mass consistent with fat, often lacking contrast enhancement except for its fibrous capsule (1, 16, 35) (Figures 1A,B). Contrast CT is particularly effective in determining fat coefficients by calculating Hounsfield units (HU) with given values between −30 to −150 HU, facilitating non-invasive diagnosis of IVLs in major venous vessels (9, 36). CT also aids in distinguishing lipomas from other soft tissue tumors based on fat density and also in identifying the presence of an intravenous thrombus by calculating the densitometry of the intraluminal mass (36, 37). CT has been performed in all reported cases of IVL except the one reported by Wahab et al. (22). This case involved the superior vena cava and right atrium and was diagnosed using only MRI and echocardiography. On MRI, IVLs appear as well-circumscribed lesions with signal characteristics similar to subcutaneous fat across all imaging sequences. Fat-specific MRI sequences appear to offer the highest specificity for diagnosing IVLs compared to other modalities confirming their fatty nature through significant loss of signal intensity on fat suppression sequences. They exhibit high signal intensity on T1-weighted images and also on T2-weighted images, although the intensity on T2-weighted images may be slightly lower compared to T1-weighted images (16, 38) (Figure 1C). However, MRI was performed in less than the half of the reported cases (2, 8, 10–12, 14, 15, 18, 21–23, 25).

### 6.2 Additional imaging modalities

In addition to standard imaging techniques, other modalities can offer valuable insights into the diagnosis and characterization of IVLs. Transthoracic and transesophageal echocardiography can provide useful tissue information, including echogenicity, calcification, vascularity, and evaluation of right atrium extension, aiding in the diagnosis of lipomas (2). Nonetheless, echocardiography was performed in only 4 of the total reported cases. Another imaging technique which may demonstrate a filling defect in the SVC or jugulosubclavian venous confluence with collateral development is the SVC venography (6, 12). Due to its invasiveness and limited value compared to CT or MRI, venography is not commonly used. Additionally, fludeoxyglucose positron emission tomography (FDG-PET) is suggested as a technique which can aid in differentiating malignant from benign lipomatous tumors and between different liposarcoma subtypes, though its use in IVLs of the head and neck has not been reported (1).

### 6.3 Histopathological analysis of IVLs

IVLs are characterized by distinctive histological features. Macroscopically, IVLs present as well-circumscribed, pale yellow, lobulated fatty masses occupying the vein lumen, which may or may not be associated with thrombus formation (5, 6). Microscopically, IVLs exhibit well-differentiated tumor predominantly composed of mature white adipose cells with non-centrally located nuclei and thin fibrous septa in some areas, encapsulated by collagenous tissue. IVLs typically lack necrosis, hemorrhage, or calcifications (14, 15).

### 6.4 Differential diagnosis

In addition to lipomas, the differential diagnosis of intravascular masses containing fat encompasses a spectrum of benign and malignant conditions such as thrombus formation, other primary tumors, and secondary tumor extension (39).

Among benign tumors, leiomyomas, originating from smooth muscle cells of the endothelium, represent the most prevalent venous tumors (3, 4). Another benign entity, fibrolipoma, characterized by neoplastic fat cells within dense collagen, has been reported, including a unique case involving the femoral vein (40). Hemangiomas, deriving from endothelial cells, have also been documented, with Hu et al. (41) reporting a rare case of a massive unicameral cardiac hemangioma associated with a persistent left SVC.

Among malignant tumors, leiomyosarcomas, arising from smooth muscle tissue of the endothelium, are the most common primary tumors of the vena cava, often leading to luminal obliteration (30). Fibrosarcomas, originating from connective tissues, and endotheliomas and hemangioendotheliomas, derived from endothelial cells, are also to be considered. Although venous angiosarcomas are rare, reported cases suggest their potential presence, characterized by mesenchymal cells with epithelioid morphology, focal nuclear atypia in adipocytes, vascular channels, and CD31 endothelial marker positivity (42). Liposarcoma, the malignant variant of lipoma, typically appear on CT as heterogeneous masses with areas of fat density interspersed with soft tissue density. They usually include thick septa and nodular or globular areas of non-adipose tissue that show heterogeneous patterns of signal intensity and enhancement due to necrosis, hemorrhage, myxoid changes or cellular areas. On T1-weighted images generally exhibit intermediate to low signal intensity and tend to show heterogenous high signal intensity on T2-weighted images. Fat-suppressed sequences and gadolinium-enhanced sequences can show heterogeneous enhancement, reflecting areas of vascularization and necrosis (35, 38). Cytogenetic analysis may play a crucial role in establishing the diagnosis (1). Head and neck intravenous location of liposarcoma has not been reported in the literature so far (26).

## 7 Management of IVLs: from observation to intervention

### 7.1 Surgical and non-surgical treatment options

Treatment guidelines for IVLs have not yet been established, most likely due to their rarity. Given that most IVLs are asymptomatic and exhibit slow growth, some authors suggest that invasive treatment may not be necessary for asymptomatic cases (2, 8–10, 13, 23, 25, 26, 36). However, regular follow-up with imaging examinations is essential to monitor any changes in the tumor over time (38). Nevertheless, other authors state that surgical resection is crucial to rule out malignancies and prevent the potential risk of thrombus formation, embolization, penetration of the right atrium and venous occlusion (2, 12, 17). This viewpoint supports the surgical removal of asymptomatic IVLs, particularly when the patient is fit for surgery and the lesion’s location allows for a safe approach without risking damage to nearby structures, thereby making the risk–benefit balance favorable in these cases (2, 12, 17, 18). This is especially recommended for large lipomas or those that are mobile, as they pose a risk for future pulmonary thromboembolism (43). Another factor endorsing this position is that, despite no reported cases of intravenous liposarcoma in the literature (26), surgical management in asymptomatic patients may be necessary to exclude malignancy in scenarios where CT or MRI cannot reliably differentiate between benign lipomas and well-differentiated liposarcomas (2), or where the lesion displays radiologic features of vascular invasiveness and association with a fatty thrombus, which are highly suggestive of malignancy. Surgical excision not only provides a definitive histological diagnosis but also prevents these risks, with no major complications reported in the performed surgeries. Furthermore, Mordant and colleagues argue that excision should be mandatory since surgical treatment is safe without the need of lifelong anticoagulation (12, 20).

Management of IVLs in other regions varies. Although no published reports exist on tumor resection for IVLs of the IVC (29, 36, 43, 44), half of the cases involving the renal vein have undergone surgery (35, 45). Additionally, all published cases of iliofemoral IVLs have been treated with surgical resection (3, 32, 40, 46–48).

### 7.2 Surgical excision techniques

The surgical approach for excising IVLs in the head and neck should be carefully considered and supported by adequate preoperative anatomical imaging. Its primary challenge is the proximity of relevant anatomical structures, therefore, when planning intervention, it is crucial to balance achieving complete tumor removal with minimizing the risk of complications and unnecessary functional and cosmetic morbidity (1).

The resection techniques described in the literature vary depending on the anatomical localization and extent of the tumor. It should also be noted that some published cases do not describe the surgical technique employed. Nonetheless, a median sternotomy combined with transcervical approach has been the most frequently used surgical approach, as it allows control of all major thoracic veins, minimizes the risk of tumor embolization, limits blood loss, and permits en bloc resection of the tumor if necessary (2, 4, 12, 16, 17, 24). Some reports describe the use of cardiopulmonary bypass to excise SVC IVLs when they extend into or are proximal to the right atrium (14–16, 18, 22).

To remove the IVL, a longitudinal (6, 16) or transverse (12) venotomy can be performed, and the tumor can then be pulled down and excised (6, 11, 18). The venous wall may be primary repaired with a running 6/0 or 7/0 polypropylene suture (4) or using a patch of polytetrafluoroethylene (24) or pericardium for surgical defect closure (2, 18). Another method for tumor removal is en bloc resection followed by end-to-end anastomosis between the two severed venous ends (12) or, in cases of IVLs isolated in the internal jugular vein, by ligation (5, 11).

Special mention is given to Cohen et al. (2), who planned a hybrid approach for an IVL extending from the jugular and subclavian vein to the SVC. They performed an endovascular technique to control the right brachiocephalic vein via the right brachial vein with a 12 mm balloon prior to venotomy to prevent the risk of tumor embolization and minimize blood loss during the en bloc excision of the IVL.

### 7.3 Post-treatment outcomes and follow-up

Among the 16 surgical patients reported in the literature, the administration of antiplatelet agents (2, 5, 21) or anticoagulants (4, 16, 18, 22) in the immediate postoperative period is noted in only 7 cases, as well as in the non-surgical case reported by Santos et al. (13), associated with sarcoidosis and long-term corticosteroid therapy.

Most cases after surgical removal had an uneventful recovery. Two postoperative complications were reported: a pulmonary embolism one week after discharge (21) and Dressler’s syndrome on postoperative day 25 (2).

Short-term follow-up imaging should be considered to assess for central vein stenosis or occlusion. Notably, many authors did not specify the surveillance regimen, and those who did typically indicated short-term follow-up with an average duration of 10 months (range 1–36 months) (2, 4, 5, 7, 12–16, 18). No evidence of tumor recurrence was observed during follow-up. Based on this, Sundaram et al. (16) suggested that due to the low incidence of tumor recurrence, long-term follow-up does not appear to be necessary.

## 8 Conclusion

IVLs represent rare benign neoplasms that grow into the venous wall. While often asymptomatic, they have the potential to induce congestive venous symptoms and venous thromboembolism. CT and MRI are the most valuable imaging techniques for confident identification of IVLs, with CT being the most frequently used throughout the literature. For symptomatic patients, surgical excision via median sternotomy and cervicotomy with subsequent venous reconstruction represents the main treatment strategy. The management of asymptomatic patients, however, remains controversial due to the limited number of reported cases. Despite this, our mini-review underscores a prevailing trend toward the surgical removal of IVLs. This inclination is driven by the necessity to accurately exclude malignancy based on cross-sectional imaging alone and to mitigate the risks of venous thromboembolism, particularly in larger or mobile lipomas. Our findings highlight that this proactive approach is substantiated by a remarkably low complication rate and the documented absence of recurrence following surgical excision. Further research is needed to accurately determine the real risk of complications and morbidity in asymptomatic IVL patients in order to establish an evidence-based approach for individual treatment selection.

## Author’s note

This article was written by members and invitees of the International Head and Neck Scientific Group (http://www.ihnsg.com).
